# Development and validation of MRI-based model for the preoperative prediction of macrotrabecular hepatocellular carcinoma subtype

**DOI:** 10.1186/s13244-022-01333-1

**Published:** 2022-12-21

**Authors:** Ismail Bilal Masokano, Yigang Pei, Juan Chen, Wenguang Liu, Simin Xie, Huaping Liu, Deyun Feng, Qiongqiong He, Wenzheng Li

**Affiliations:** 1 Department of Radiology, Xiangya Hospital, Central South University, No. 168 Xiangya Road, Kaifu District, Changsha, 410008 Hunan China; 2 National Clinical Research Center for Geriatric Disorders, Xiangya Hospital, Central South University, Changsha, 410008 Hunan China; 3 Department of Radiology, The Third Xiangya Hospital, Central South University, Changsha, 410013 Hunan China; 4 Department of Pathology, Xiangya Hospital, Central South University, Changsha, 410008 Hunan China

**Keywords:** Macrotrabecular hepatocellular carcinoma, Magnetic resonance imaging, Nomograms, Decision-making

## Abstract

**Background:**

Macrotrabecular hepatocellular carcinoma (MTHCC) has a poor prognosis and is difficult to diagnose preoperatively. The purpose is to build and validate MRI-based models to predict the MTHCC subtype.

**Methods:**

Two hundred eight patients with confirmed HCC were enrolled. Three models (model 1: clinicoradiologic model; model 2: fusion radiomics signature; model 3: combined model 1 and model 2) were built based on their clinical data and MR images to predict MTHCC in training and validation cohorts. The performance of the models was assessed using the area under the curve (AUC). The clinical utility of the models was estimated by decision curve analysis (DCA). A nomogram was constructed, and its calibration was evaluated.

**Results:**

Model 1 is easier to build than models 2 and 3, with a good AUC of 0.773 (95% CI 0.696–0.838) and 0.801 (95% CI 0.681–0.891) in predicting MTHCC in training and validation cohorts, respectively. It performed slightly superior to model 2 in both training (AUC 0.747; 95% CI 0.689–0.806; *p* = 0.548) and validation (AUC 0.718; 95% CI 0.618–0.810; *p* = 0.089) cohorts and was similar to model 3 in the validation (AUC 0.866; 95% CI 0.801–0.928; *p* = 0.321) but inferior in the training (AUC 0.889; 95% CI 0.851–0.926; *p* = 0.001) cohorts. The DCA of model 1 had a higher net benefit than the treat-all and treat-none strategy at a threshold probability of 10%. The calibration curves of model 1 closely aligned with the true MTHCC rates in the training (*p* = 0.355) and validation sets (*p* = 0.364).

**Conclusion:**

The clinicoradiologic model has a good performance in diagnosing MTHCC, and it is simpler and easier to implement, making it a valuable tool for pretherapeutic decision-making in patients.

**Supplementary Information:**

The online version contains supplementary material available at 10.1186/s13244-022-01333-1.

## Key points

- Clinicoradiologic model has a good performance in diagnosing MTHCC.
- In validation set, models 2 and 3 did not significantly improve predictive performance compared to clinicoradiologic model.
- Clinicoradiologic model yielded higher clinical benefit at MTHCC threshold probability of 10%.
- Clinicoradiologic model is as beneficial as model 3, yet simpler and easier.

**Supplementary Information:**

The online version contains supplementary material available at 10.1186/s13244-022-01333-1.

## Background

Being the most common primary liver malignancy and second-most deadly cancer [1], the precise preoperative diagnosis of hepatocellular carcinoma (HCC) has been increasingly investigated for proper management and improvement in patients' quality of life. Pretherapeutic identification of the tumor subtype is essential because different HCC subtypes respond differently to therapies due to distinct genetic, clinical, and prognostic characteristics [2, 3].

Macrotrabecular hepatocellular carcinoma (MTHCC) is a peculiar subtype with high genetic alterations, including gene mutations (TP53 mutations), amplifications (FGF19 gene), and overexpression of pro-angiogenesis proteins (ESM1, angiopoietin, and endothelial growth factors, etc.) [3–5]. Clinically, patients with MTHCC have higher serum *α*-fetoprotein (AFP), larger and more aggressive lesions, higher recurrence rates, and poorer survival than patients with other subtypes [3, 6].

Therefore, an accurate diagnosis of MTHCC can assist in effective clinical decision-making to achieve personalized patient care. Currently, pathologic examination of HCC specimens is the goal standard for diagnosing histologic subtypes. Though the subtype of many HCCs can be identified on biopsy, a routine preoperative biopsy is controversial, and the clinical risk–benefit balance remains unclear [7]. A biopsy is associated with sampling bias from tumor heterogeneity [8] because highly heterogenous lesions are characterized by varying proportions of necrotic and regressive changes, which may make sampled specimens inadequate for pathologic examination and often warrant a repeat biopsy [9]. Consequently, given the risk of the need for a repeat sampling, inadvertent hemorrhage, and tumor embolization [10], a biopsy may be unsuitable for routine preoperative HCC subtype identification. Thus, a noninvasive approach that uses CT or MRI before surgery to identify the MTHCC is crucial in patients' prognostication.

Presently, a few studies have described some MRI features of MTHCC. Mule et al. identified substantial necrosis on MRI as an independent predictor of the MTHCC subtype [11]. Additionally, Cannella et al. identified a larger tumor size and the presence of tumor-in-vein [12], Liang et al. [13] found the absence of enhancing capsule and blood products in the lesion, and Rhee et al. [14] showed arterial phase peritumoral enhancement (corona enhancement), intratumoral arteries, and rough tumor margin to be significantly associated with the MTHCC subtype on MR imaging. However, these studies were based on the qualitative evaluation of MR or CT-based imaging findings without incorporating quantitative texture features.

Radiomics is a noninvasive approach that can quantitatively and more objectively identify the histologic characteristics of a tumor by evaluating the grayscale differences in an image. Thus, radiomics can potentially aid in the preoperative identification of the macrotrabecular hepatocellular subtype. To our knowledge, only one study [15] on the quantitative assessment of HCC texture features for preoperative prediction of the MTHCC subtype has been published—it was limited by a small sample size (32 MTHCC) and the lack of a validation cohort.

Therefore, the aim of this study was to build and validate three models (clinicoradiologic model, fusion radiomics signature, and combined radiomics model) to predict MTHCC noninvasively, assess their clinical utility, and decide on the suitable model that will assist in patient management.

## Materials and methods

### Patients' enrollment

The Medical Ethics Committee of our institution (Xiangya Hospital, Central South University) approved this retrospective study (Approval Number: 2018111101) and waived the requirement for patient consent.

**Fig. 1:**
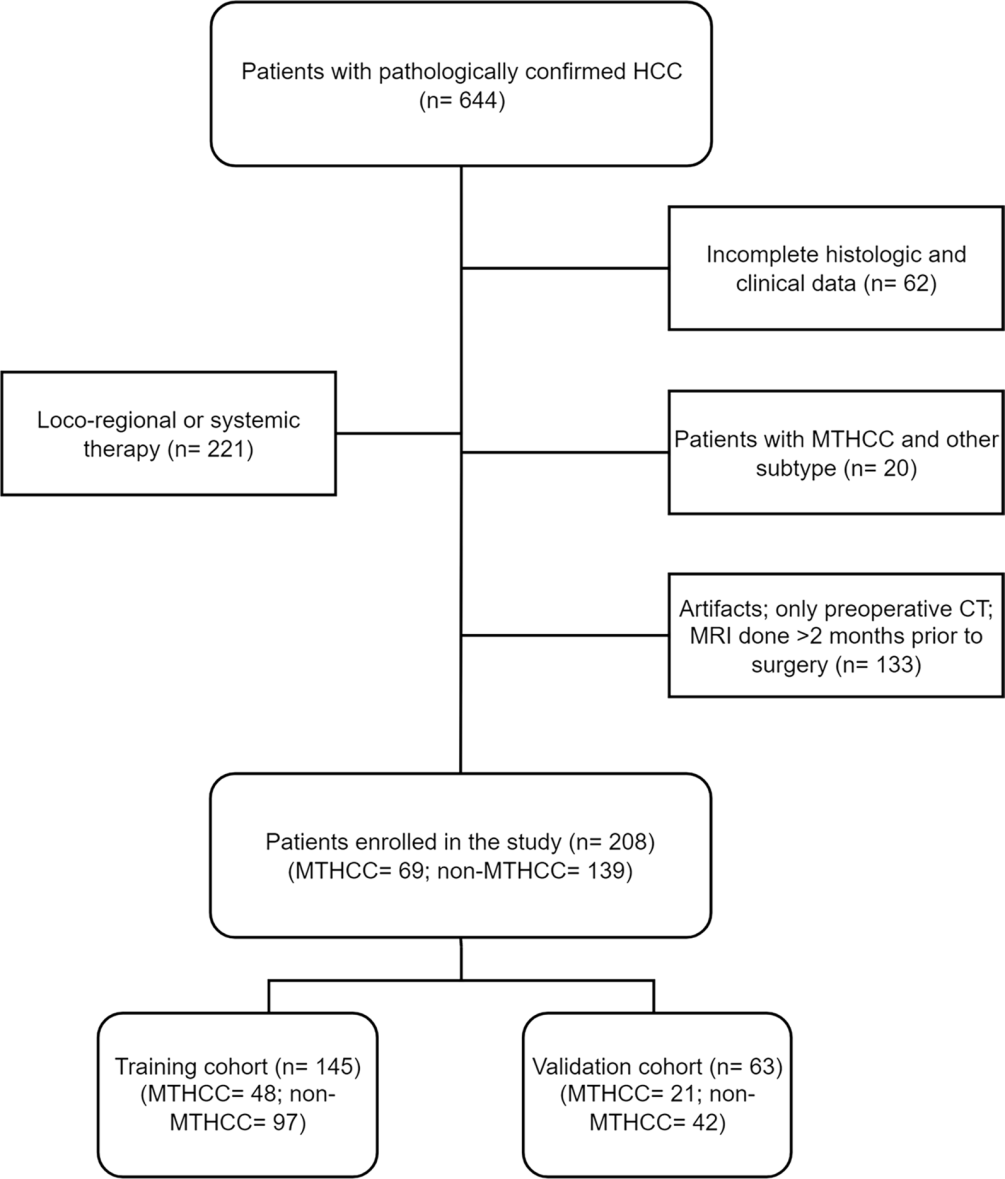
Flow diagram of patient enrollment

We searched the database of our institution to retrieve the pathologic, radiologic, and clinical data of patients with histologically confirmed hepatocellular carcinoma who had hepatectomy between July 2017 and July 2020. Consecutive patients with pathologically confirmed HCC were identified and enrolled based on the inclusion criteria: (1) histologically confirmed HCC with a detailed pathologic report; (2) MR imaging performed within two months before surgery; and (3) sufficient image quality to allow accurate interpretation of radiologic features. Patients were excluded if they fell within one or more of the following: (1) unavailability of MR imaging or imaging was done in another institution; (2) suboptimal image quality making the evaluation of imaging characteristics difficult; (3) previous intervention therapy (LRT) or systemic chemotherapy in new HCC patients and those with recurrence; and (4) presence of an MTHCC and another subtype in the same patient. A total of 208 patients out of the 644 subjects with HCC were enrolled and randomly split into training and validation cohorts in a ratio of 7:3 using the non-exhaustive tenfold cross-validation (Fig. 1).

### Histologic characteristics

**Fig. 2:**
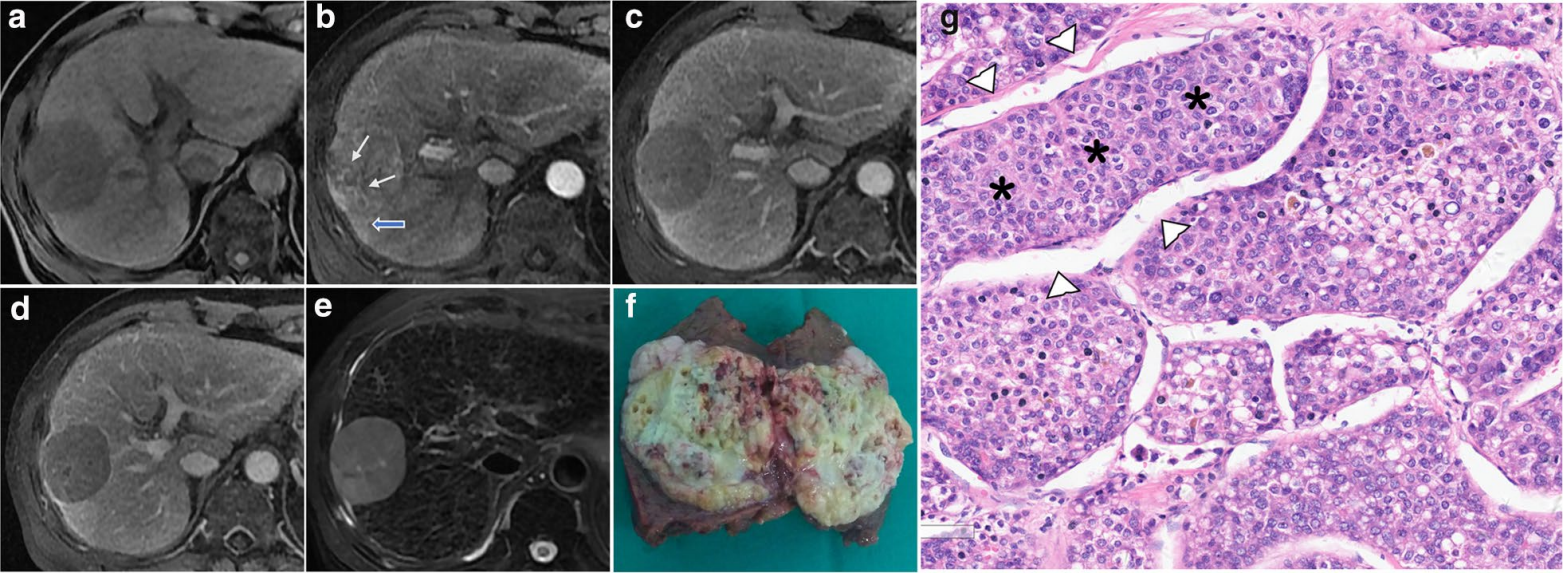
MR Images (**a**–**e**) in a 73-year-old man with a 51-mm MTHCC. The lesion shows a non-rim arterial phase hyperenhancement with intratumoral arteries (white arrows) and wedge-shaped arterial corona enhancement (blue arrows) (**b**); non-peripheral washout and enhancing capsule in the portal and delayed phases (**c** and **d**). The gross appearance of the lesion after resection (**f**) and microscopic examination (**g**) showing thick sheets of hepatocytes (*) surrounded by vascular spaces (arrowheads). (Hematoxylin & Eosin, magnification×200). The criterion for the pathologic diagnosis of MTHCC is a hepatocellular carcinoma with a predominant macrotrabecular architectural pattern (more than 6 cells thick) involving more than 50% of the tumor [3]

The hepatectomy specimens were reviewed independently by two pathologists (D.F. and QQ.H. with 20 and 15 years of experience, respectively) who were blinded to the patients' clinical data. The following histologic characteristics were recorded: tumor differentiation based on Edmondson-Steiner grade (the predominant grade was allocated to lesions showing different grades); histologic subtype including MTHCC (Fig. 2) [3]; microvascular invasion, and satellite nodules. Discrepancies in assessments were resolved by consensus.

### MR image acquisition

All patients had MR imaging using a 3.0T MR scanner (Discovery MR750w, GE Healthcare or Siemens Healthcare). The public protocol was adopted, and the imaging parameters are shown in detail in Table 1. One hundred and sixty-one patients had MRI with an extracellular contrast agent: 15 ml of gadodiamide (Omniscan, GE Healthcare) was injected at a rate of 0.2 ml/kg, while 47 had imaging with a hepatobiliary agent: 10 ml of gadoxetic acid disodium (Primovist; Bayer-Global) was injected, followed by 20 ml of 0.9% saline at an injection rate of 1 ml/s or 2 ml/s. Following the pre-contrast images, contrast-enhanced dynamic images and hepatobiliary phase images were acquired. The arterial phase (AP), portal venous phase (PVP), delayed phase or transitional phase (for gadoxetic acid disodium agent), and hepatobiliary phases (HBP) were acquired within 25–30 s, 65–75 s, 130–150 s, and 15–20 min, respectively, after the contrast injection.

**Table 1:** Imaging parameters

Parameters	TR (msec)	TE (msec)	BW (Hz/pixel)	FOV (mm)	Matrix	Slice thickness (mm)	NEX	Flip angle (°)	Contrast agents used
Respiratory-triggered FS T2WI	3529	80.3	83.33	36	320 × 320	6.0	2	110	15 ml of gadodiamide (Omniscan, GE Healthcare) in 161 patients
Breath-hold axial LAVA-flex T1WI	2.7	1.3	142.86	40	512 × 512	4.0	1.0	12	10 ml of gadoxetic acid disodium (Primovist; Bayer-Global) in 47 patients
Breath-hold VIBE T1WI	2.7	1.3	1040	40	320 × 167	3.0	2.0	9	

*TR* repetition time, *TE* echo time, *BW* bandwidth, *FOV* field of view, *NEX* number of excitations, *FS* fat saturated, *LAVA* Liver Imaging with Volume Acceleration-flexible

### Model 1

#### Patients' clinical information

The clinical data were obtained: gender, age at surgery, hepatitis B (HBV) and hepatitis C virus (HCV) status, immediate preoperative AFP, albumin, total bilirubin, aspartate transaminase (AST), and alanine transaminase (ALT) levels (Table 2).

#### Qualitative MR image analysis

Blinded to clinical and histopathologic information, all patients' MR images were retrospectively reviewed. Two readers (P.Y. and L.W., with 10 and 15 years of experience in abdominal imaging, respectively) independently interpreted the MRI features: (1) number and diameter of tumors; (2) tumor necrosis [11]; (3) intratumor arteries [16]; (4) corona enhancement [17]; (5) tumor heterogeneity [18]; (6) radiologic capsule [19]; (7) intratumor hemorrhage; (8) intratumor fat; (9) characteristic late arterial phase hyperenhancement and non-peripheral washout as defined by the LI-RADS 2018 [20]; and (10) rim-like arterial phase enhancement [21]. Additional file 1: Appendix E1 and Figs. S1–4 provide a detailed description of the previous features.

#### Construction of model 1

Univariate analysis was used to compare the differences in clinical factors and qualitative MRI features between MTHCC and non-MTHCC. A *p* value < 0.05 indicates a significant difference. The significant predictors in the training set were entered into a logistic regression analysis to build a clinicoradiologic model (model 1). The odds ratio (OR) and 95% confidence intervals (CI) for each independent predictor were computed.

### Model 2

#### Tumor segmentation

The ITK-SNAP software (Version 3.8.0) was used to manually segment the entire tumor from T2W, AP, and PVP images as previously reported by studies [11, 14] on the imaging differences between MTHCC and non-MTHCC. Two readers (I.B. and C.J., with 4 years of experience in abdominal imaging) performed the segmentation. Only the largest lesion was included in patients with multiple tumors (the lesion with a clearer margin was used in patients with equally sized lesions). All patients with multiple lesions in the MTHCC group have only MTHCCs, while those in the non-MTHCC group have non-MTHCCs. The whole tumor was segmented slice by slice by outlining the contour 1–2 mm within its boundary and avoiding major vessels and adjacent liver parenchyma. The tumor in the last slices was not included to avoid volume averaging with adjacent structures. Initially, both readers independently segmented 50 randomly selected tumors (including 36 MTHCCs and 14 non-MTHCCs). After that, a reader (I.B.) repeated the same segmentation on the 50 tumors a week later to obtain intra- and inter-rater intra-class correlation coefficients (ICC) as described by [22]. Texture features with ICC > 0.75 were considered to have a good agreement. Reader 1 (I.B.) continued with the remaining image segmentation.

#### Feature extraction

The reader (I.B.) transferred the ROIs into the radiomics platform of AK software 3.3.0 (Analysis kit, GE Healthcare). The MR images and segmented ROIs were first preprocessed. Tumors and ROIs were compared side by side to ensure all ROIs match exactly with their corresponding tumors. Images were resampled using a 1.0 mm voxel size along the X, Y, and Z coordinates, respectively. A Gaussian filter of 0.5 mm bandwidth was used to filter noise from the images. First-, second-, and higher-order features were extracted. A total of 3111 features—1037 features from each phase—were extracted (Additional file 1: Table S1).

To select the robust radiomics features, the features with outliers (under the first quartile or above the third quartile of the feature distribution) and missing values were replaced by the feature’s median value in the dataset. Finally, all T2W, AP, and PVP features were standardized using zero-mean normalization to remove pixels that fall outside a specified range of gray levels.

#### Construction of model 2

First, the radiomics features with ICC > 0.75 from the T2W, AP, and PVP images in the training cohort were selected to train the predictive model. Subsequently, potential features that were significantly different between the MTHCC and non-MTHCC groups were obtained using the Mann–Whitney *U* test. Thereafter, the features were entered into the least absolute shrinkage and selection operator (LASSO) regression model to select the most valuable features by tuning the hyperparameter λ with the smallest tenfold cross-validation error in the training set for each set of the images. Model 2 (fusion radiomics signature) was thus built using the combined robust features from T2W, AP, and PVP images.

The radiomics score for each patient was calculated based on the formula in Additional file 1: Appendix E2.

#### Construction and validation of model 3

**Fig. 3:**
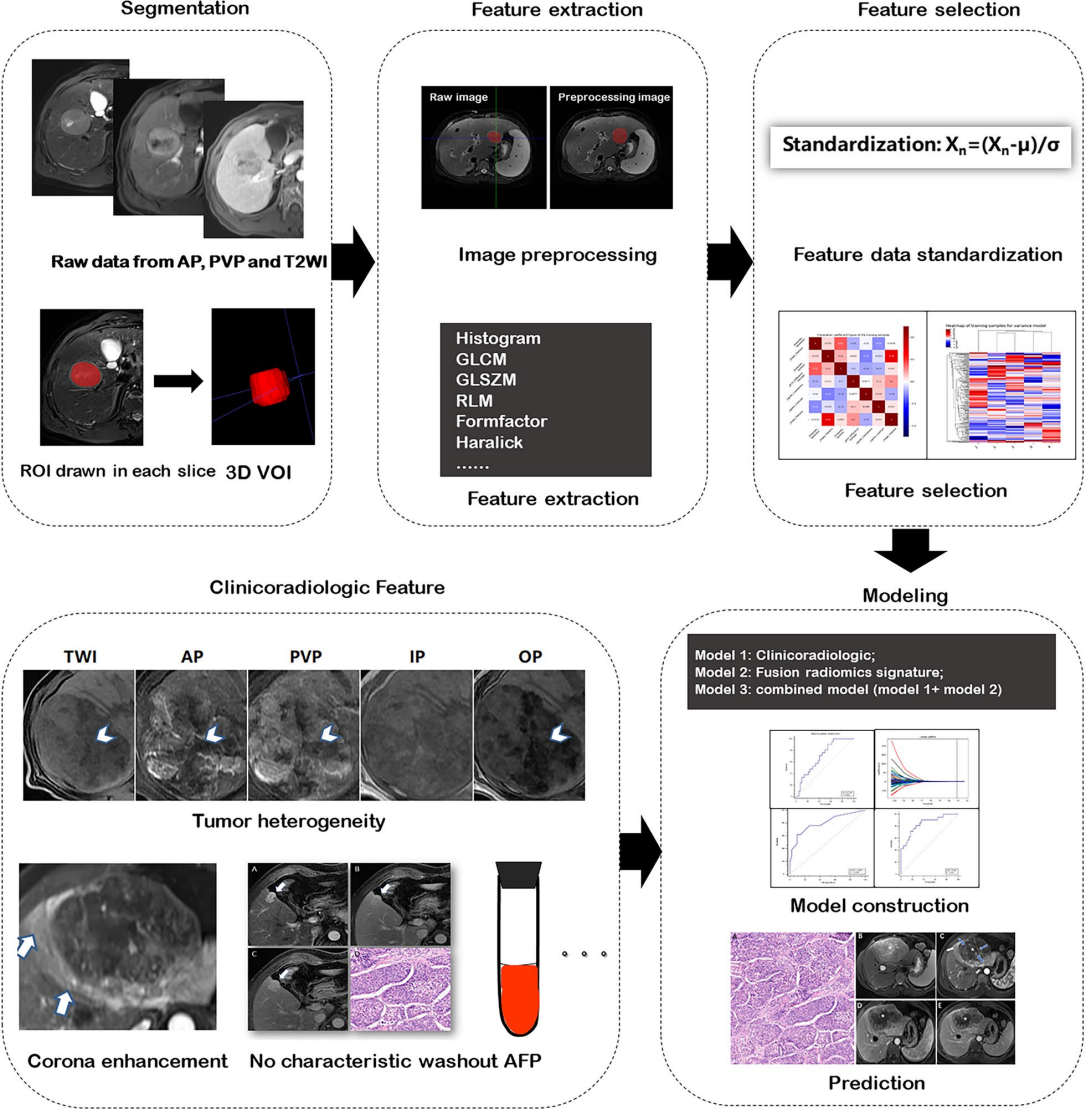
Workflow of model construction

Variables from model 1 and model 2 were entered into a logistic regression to form a combined radiomics model (model 3) to predict MTHCC. The workflow of model construction is shown in Fig. 3. The predictive performance of the models (models 1, 2, and 3) in training and validation cohorts was decided by the maximum area under the curve (AUC) and the associated cutoff according to the Youden index.

#### Estimating the clinical utility of the models

A decision curve analysis (DCA) was used to estimate the clinical utility of the models by calculating the net benefits for a range of threshold probabilities (percentage risk threshold of detecting the subtype). For each decision curve, the net clinical benefit was computed using the following formula [23]:

$${\text{Net}}\, {\text{benefit }} = \frac{{{\text{True }}\,{\text{positives}}}}{N} - \frac{{{\text{False}}\,{\text{ positives}}}}{N} \times \frac{{p_{t} }}{{1 - p_{t} }}$$

where *p*_*t*_ is the threshold probability for detecting a positive patient.

The decision curve plots net clinical benefit (y-axis) against threshold probability (x-axis). The clinical utility of the curve is indicated by the highest net clinical benefit at the lowest threshold probability.

### Nomogram construction

A nomogram for the most clinically applicable model was constructed based on the AUC performance and clinical utility at the lowest threshold probability. The process of graphical presentation of the nomogram is described in Additional file 1: Appendix E3.

### Statistical analysis

Statistical analyses were performed using IBM SPSS Statistics (version 25, IBM SPSS Inc.) and R statistical software (Version 3.5.1). Univariate analyses were used to compare differences between MTHCC and non-MTHCC patients regarding clinicopathologic characteristics: using chi-square or Fisher’s exact tests for categorical variables and Mann–Whitney *U* test for continuous variables. Continuous variables were summarized as median and interquartile range (IQR). Cohen's kappa coefficient was used to evaluate the inter-reader agreement for each qualitative MRI feature. Intra- and inter-rater intra-class correlation coefficients (ICC) were obtained to evaluate the reliability of the manual segmentation [24]. The diagnostic performance of the models 1, 2, and 3 in differentiating MTHCC from non-MTHCC was assessed by the AUC (with 95% CI), sensitivity, specificity, and accuracy in training and validation cohorts. A model is considered to have excellent, good, or poor performance when it has an AUC of 0.85–1.0, 0.7–0.85, or < 0.7, respectively [25]. The curves of the models were compared using the Delong test.

The receiver operating characteristic (ROC) curves were plotted using MedCalc software (Version 19.5.0). The “glmnet,” “rms,” and “dca” packages were used to perform LASSO regression, nomogram construction, and DCA on R, respectively. A two-tailed *p* value < 0.05 indicated statistical significance.

## Results

### Clinical and imaging characteristics

A total of 145 patients (median age, 53 years; interquartile range (IQR), 45–62 years; 21 women and 124 men; 48 MTHCC and 97 non-MTHCC) were included in the training cohort and 63 patients (median age, 52 years; IQR 41–62 years; 5 women and 58 men; 21 MTHCC and 42 non-MTHCC) were included in the validation cohort (Fig. 1). Sixty-nine of the 208 HCC patients had the MTHCC subtype (33.2%); proportions of other subtypes in our patients are presented in Additional file 1: Fig. S5A.

Clinical, pathologic, and imaging variables were not significantly different between the training and validation cohorts (*p* = 0.087–0.97). By univariate analysis, MTHCC significantly differed from non-MTHCC in the levels of serum AFP, tumor grade, tumor capsule, heterogeneity, intratumoral arteries, corona enhancement, and absence of washout (all *p* < 0.05). Tumor grade and intratumoral arteries were not significantly different in the two groups in the validation cohort (Tables 2 and 3).

**Table 2:** Demographic, clinical, and pathologic characteristics of 208 patients according to MTHCC subtype

Variables	Training set (*n* = 145) *n* (%) or median (IQR)				Validation set (*n* = 63) *n* (%) or median (IQR)			
	Available data	MTHCC (*n* = 48)	Non-MTHCC (*n* = 97)	*p* value	Available data	MTHCC (*n* = 21)	Non-MTHCC (*n* = 42)	*p* value
Gender	145			0.081	63			0.510
Male	124 (85.5)	43 (89.6)	81 (83.5)		58 (92.1)	20 (95.2)	30 (90.5)	
Female	21 (14.5)	5 (10.4)	16 (16.5)		5 (7.9)	1 (4.8)	4 (9.5)	
Age	145	51 (45.50–59.75)	53 (45–63)	0.380	63	44 (40–50.5)	55.5 (44.5–64)	0.026*
AFP	145			0.005*	63			0.037^*^
< 20 ng/ml	58 (40.0)	12 (25.0)	46 (47.4)		30 (47.6)	6 (28.6)	24 (57.1)	
20–400 ng/ml	35 (24.1)	12 (25.0)	23 (23.7)		14 (22.2)	6 (28.6)	9 (19.0)	
> 400 ng/ml	52 (35.9)	24 (50.0)	28 (28.9)		19 (30.2)	9 (42.9)	10 (23.8)	
ALT	145			0.674	63			0.157
< 40U/l	81 (55.9)	28 (58.3)	53 (54.6)		32 (50.8)	8 (38.1)	24 (57.1)	
> 40U/l	64 (44.1)	20 (41.7)	53 (45.4)		31 (49.2)	13 (61.6)	18 (42.9)	
AST	145			0.521	63			0.374
< 35U/l	64 (44.1)	23 (47.9)	41 (42.3)		28 (44.4)	11 (52.4)	17 (40.5)	
> 35U/l	81 (55.9)	25 (52.1)	56 (57.7)		35 (55.6)	10 (47.6)	25 (59.5)	
Albumin	145			0.262	63			0.216
< 40U/l	67 (46.2)	19 (39.6)	48 (49.5)		31 (49.2)	8 (38.1)	23 (58.4)	
> 40U/l	78 (53.8)	29 (60.4)	49 (50.5)		32 (50.8)	13 (61.9)	19 (45.2)	
Cirrhosis	145			0.193	63			0.807
Present	128 (88.3)	88 (90.7)	40 (83.3)		53 (84.1)	18 (85.7)	35 (83.3)	
Absent	17 (11.7)	8 (16.7)	9 (9.3)		10 (15.9)	3 (14.3)	7 (16.7)	
HBV	145			0.395	63			0.284
Present	109 (75.2)	34 (70.8)	75 (77.3)		49 (77.8)	18 (85.7)	31 (73.8)	
Absent	36 (24.8)	14 (29.2)	22 (22.7)		14 (22.2)	3 (14.3)	11 (26.2)	
HCV	145			0.093	63			1.000
Present	6 (4.1)	4 (8.3)	2 (2.1)		3 (4.8)	1 (4.8)	2 (4.8)	
Absent	139 (95.9)	44 (91.7)	95 (97.9)		60 (95.5)	20 (95.2)	40 (95.2)	
Total bilirubin	145			0.880	63			0.352
< 20.4 µmol/l	126 (86.9)	42 (87.5)	84 (86.6)		52 (82.5)	16 (76.2)	36 (85.7)	
> 20.4 µmol/l	19 (13.1)	6 (12.5)	13 (13.4)		11 (17.5)	5 (23.8)	36 (14.3)	
Pathologic data								
Edmondson-Steiner grade	145			0.029^*^	63			0.484
Grade I	13 (9)	0	13 (13.4)		6 (9.5)	1 (4.8)	5 (11.9)	
Grade II	102 (70.3)	37 (77.1)	65 (67)		46 (73)	16 (76)	30 (71.4)	
Grade III	30 (20.7)	11 (22.9)	19 (19.6)		11 (17.5)	4 (19)	7 (16.7)	
MVI	145			0.216	63			0.285
Present	74 (41)	28 (58.3)	46 (47.4)		30 (47.6)	12 (57.1)	18 (42.9)	
Absent	71 (49)	20 (41.7)	51 (52.6)		33 (52.4)	9 (42.9)	24 (57.1)	
Satellite nodules	145			0.265	63			0.530
Present	37 (25.5)	15 (31.3)	22 (22.7)		15 (23.8)	6 (26.6)	9 (21.4)	
Absent	108 (74.5)	33 (68.8)	75 (77.3)		48 (76.2)	15 (71.4)	33 (78.6)	

*MTHCC* macrotrabecular hepatocellular carcinoma, *IQR* interquartile range, AFP alpha-fetoprotein, *AST* aspartate transaminase, *ALT* alanine transaminase, *HBV* hepatitis B virus (hepatitis B surface antigen), *HCV* hepatitis C virus (hepatitis C antigen), *MVI* microvascular invasion

*Significant *p* values (*p* < 0.05)

**Table 3:** MR imaging characteristics of the HCC lesions according to MTHCC subtype

Image traits	*n* (%) or median (IQR) Training set (*n* = 145)				*n* (%) or median (IQR) Validation set (*n* = 63)				
	Available data	MTHCC (*n* = 48)	Non-MTHCC (*n* = 97)	*p* value	Available data	MTHCC (*n* = 21)	Non-MTHCC (*n* = 42)	*p* value	Interrater agreement**
Number of patients	145			0.251	63			0.513	1.00
Patients with single lesions	116 (80)	41 (85.4)	75 (77.3)		51 (81)	16 (76.2)	35 (83.3)		
Patients with multiple lesions	29 (20)	7 (14.6)	22 (22.7)		12 (19)	5 (23.8)	7 (16.7)		
Tumor size* (mm)	145	48.2 (32.05–91.45)	50.1 (29.65–73.75)	0.925	63	34.7 (24.2–72.25)	48.7 (25.05–67.7)	0.435	0.99
Tumor capsule	145			0.014^a^	63			0.001^a^	0.96
Absent	29 (20)	14 (29.2)	15 (15.5)		12 (19)	6 (28.6)	6 (14.3)		
Partial	20 (13.8)	9 (18.8)	11 (11.3)		8 (12.7)	7 (33.3)	1 (2.4)		
Complete	96 (66.2)	25 (52.1)	71 (73.2)		43 (68.3)	8 (38.1)	35 (83.3)		
Tumor heterogeneity	145	14 (33.3)	40 (41.2)	< 0.001^a^	63			0.001^a^	0.78
Homogenous	108 (74.5)	25 (52.1)	83 (85.6)		40 (63.5)	7 (33.3)	33 (78.6)		
Heterogeneous	37 (25.5)	23 (47.9)	14 (14.4)		23 (36.5)	14 (66.7)	9 (21.4)		
Tumor necrosis	145			0.881	63			0.332	0.97
Absent	64 (44.1)	20 (41.7)	44 (45.4)		30 (47.6)	8 (38.1)	22 (52.4)		
Present (< 20%)	28 (19.3)	11 (22.9)	17 (17.5)		15 (23.8)	6 (28.6)	9 (21.4)		
Necrosis > 20%	53 (36.6)	17 (35.4)	36 (37.1)		18 (28.6)	7 (33.3)	11 (26.2)		0.83
Intratumor arteries	145			0.047^a^	63			0.103	0.93
Present	42 (29)	19 (39.6)	23 (23.7)		13 (20.6)	7 (33.3)	6 (14.3)		
Absent	103 (71)	29 (60.4)	74 (76.3)		50 (79.4)	14 (66.7)	36 (85.7)		
Intratumor hemorrhage	145			0.633	63			0.237	0.80
Present	43 (29.7)	13 (27.1)	30 (30.9)		18 (28.6)	4 (19)	14 (33.3)		
Absent	102 (70.3)	35 (72.9)	67 (69.1)		45 (71.4)	17 (81)	28 (66.7)		
Intratumor fat	145			0.304	63			0.023^a^	0.74
Present	21 (14.5)	9 (18.8)	12 (12.4)		9 (14.3)	0	9 (21.4)		
Absent	124 (85.5)	39 (81.3)	85 (87.5)		54 (85.7)	21 (100)	33 (78.6)		
APHE	145			0.228	63			0.255	0.92
Absent	19 (13.1)	4 (8.3)	15 (15.5)		5 (7.9)	1 (4.8)	4 (9.5)		
EAPHE	11 (7.6)	2 (4.2)	9 (9.3)		4 (6.3)	0	4 (9.5)		
LAPHE	115 (79.3)	42 (87.5)	73 (75.3)		54 (85.7)	20 (95.2)	34 (81)		
Rim APHE	12 (8.3)	6 (12.5)	6 (6.3)	0.212	5 (7.9)	3 (14.3)	2 (4.8%)	0.323	
Corona enhancement	145			< 0.001^a^	63			0.036^a^	0.84
Present	23 (15.9)	17 (35.4)	6 (6.2)		7 (11.1)	5 (23.8)	2 (4.8)		
Absent	122 (84.1)	31 (64.6)	91 (93.8)		56 (88.9)	16 (76.2)	40 (95.2)		
Washout	145			< 0.001^a^	63			0.039^a^	0.83
Present	130 (89.7)	36 (75.0)	94 (96.9)		58 (92.1)	17 (81.0)	41 (97.6)		
Absent	15 (10.3)	12 (25.0)	3 (3.1)		5 (7.9)	4 (19.0)	1 (2.4)		

*MTHCC* macrotrabecular hepatocellular carcinoma, *APHE* arterial phase enhancement, *EAPHE* early arterial phase enhancement, *LAPHE* late arterial phase enhancement, *AP* arterial phase

*Tumor size based on the largest axial diameter

**Cohen’s kappa statistic and interclass correlation coefficient for qualitative and quantitative variables, respectively; *n* = number

^a^Significant *p* values (*p* < 0.05)

### Performance of model 1

Higher serum AFP > 400 ng/ml (OR 2.196; 95% CI 1.245–3.872; *p* = 0.007), more heterogeneity (OR 3.269; 95% CI 1.142–9.359; *p* = 0.027), corona enhancement (OR 3.985; 95% CI 1.217–13.050; *p* = 0.022), and absence of the washout (OR 7.773; 95% CI 1.596–37.847; *p* = 0.011) remained independent predictors of MTHCC.

The model had a good predictive performance in the training (AUC 0.773; 95% CI 0.696–0.838; sensitivity: 0.521; specificity: 0.910; accuracy: 0.722) and validation (AUC: 0.801; 95% CI 0.681–0.891; sensitivity: 0.619; specificity: 0.904; accuracy: 0.702) cohorts, respectively (Table 4).

**Table 4:** Predictive performance of models 1, 2, and 3

Model	Training cohort						Validation cohort					
	AUC (95% CI)	Sen	Spec	Accu	Cutoff	*p* value	AUC (95% CI)	Sens	Spec	Accu	Cutoff	*p* value
Model 1	0.773 (0.696–0.838)	0.521	0.910	0.722	0.438	Model 1 vs Model 2:0.548	0.801 (0.681–0.891)	0.619	0.904	0.702	0.524	Model 1 versus Model 2:0.089
Model 2	0.747 (0.689–0.806)	0.938	0.515	0.727	0.484		0.718 (0.618–0.810)	0.857	0.500	0.679	0.357	
AP	0.706 (0.642–0.766)	0.526	0.742	0.634	0.351	Model 1 vs Model 3: 0.001	0.589 (0.486–0.699)	0.571	0.595	0.583	0.381	Model 1 versus Model 3: 0.321
PVP	0.662 (0.600–0.731)	0.557	0.629	0.593	0.268	Model 2 vs Model 3:0.001	0.638 (0.538–0.738)	0.643	0.571	0.607	0.381	Model 2 versus Model 3:0.031
T2W	0.673 (0.610–0.736)	0.722	0.608	0.665	0.340		0.580 (0.470–0.681)	0.548	0.524	0.536	0.357	
Model 3	0.889 (0.851–0.926)	0.866	0.784	0.825	0.648		0.866 (0.801–0.928)	0.881	0.714	0.798	0.548	

*AP* arterial phase, *PVP* portal venous phase, *T2W* T2-weighted image, *AUC* area under the curve, *Sen* sensitivity, *Spec* specificity, *Accu* accuracy, *CI* confidence interval

### Performance of model 2

A total of 1585 texture features with intra- and inter-correlation coefficients > 0.75 (951 features from T2W images, 380 from AP, and 253 from PVP) were selected for further analysis. One hundred and eighty features significantly differed between MTHCC and non-MTHCC with ANOVA (*p* < 0.05). And 5 optimal features were selected by LASSO, and logistic regression identified four features (AP-derived glcm_Contrast, PVP-based skewness, and T2W images-based gldm_Dependence Variance and glszm_Small AreaEmphasis) associated with MTHCC (Table 5). Radiomics scores based on the above four features are presented in Additional file 1: Appendix E4 and, Figs. S5B and C.

**Table 5:** Correlation coefficients and *p* values for features after LASSO

Variables	Coef.	St. Err	*p*
Intercept	− 0.0142	0.1580	0.929
Original_glcm_Contrast [AP]	0.5061	0.1775	0.004*
Original_shape_Flatness [AP]	0.3152	0.1643	0.055
Original_firstorder_Skewness [PVP]	0.6996	0.1792	< 0.001*
Original_gldm_DependenceVariance [T2WI]	0.3312	0.1666	0.047*
Original_glszm_SmallAreaEmphasis [T2WI]	− 0.3529	0.1706	0.039*

Standard error (Std.Err); Coefficient (Coef.)

*Significant *p* value (*p* < 0.05)

**Fig. 4:**
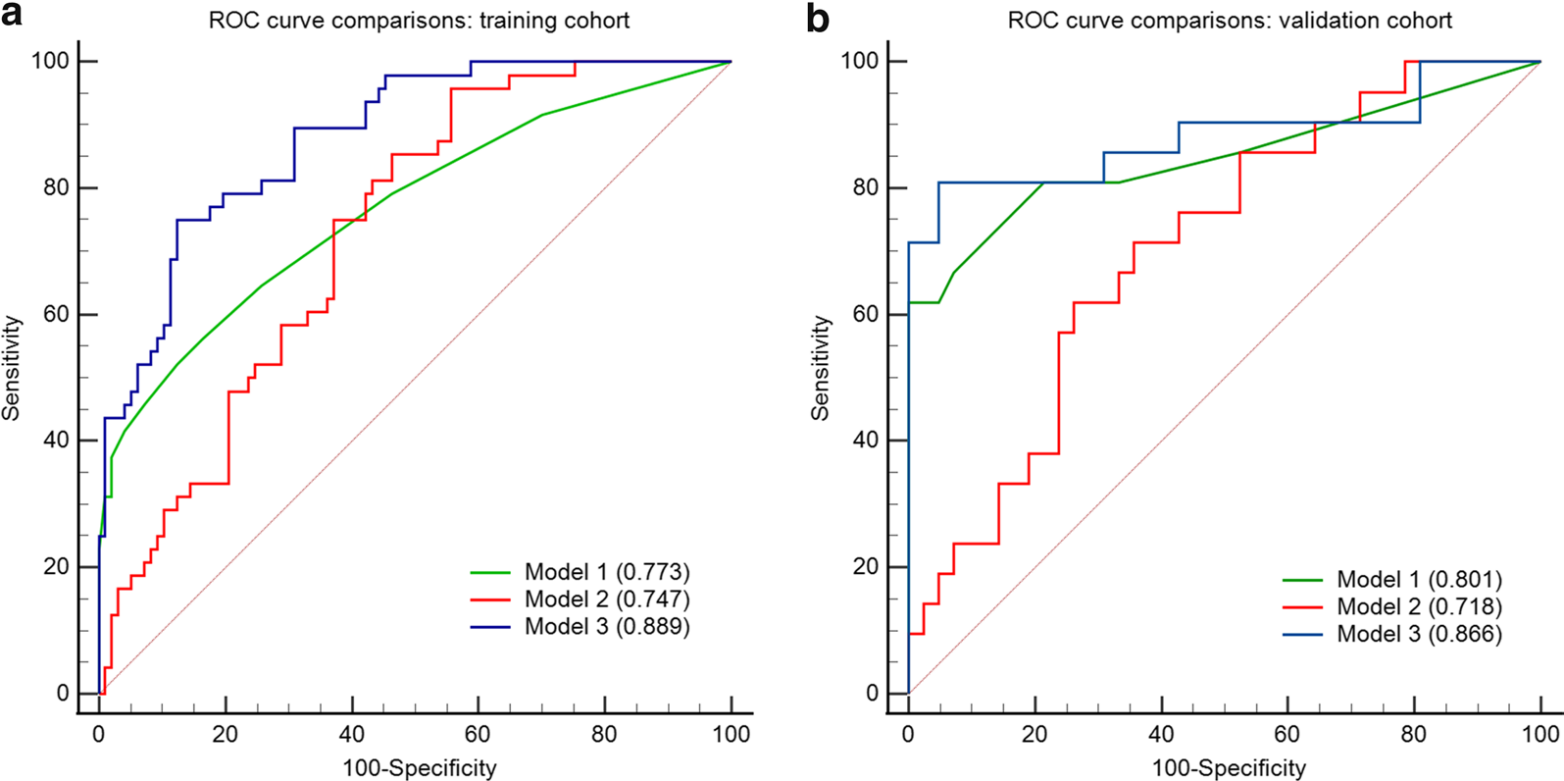
Comparison of receiver operating characteristics curves of the three models in training (**a**) and validation (**b**) cohorts

The AUCs of T2W, AP, PVP images, and model 2 are shown in Table 4. Model 2 showed a good predictive performance in the training (AUC 0.747; 95% CI 0.689–0.806; sensitivity: 0.938; specificity: 0.515; accuracy: 0.727) and validation (AUC 0.718; 95% CI 0.618–0.810; sensitivity: 0.857, specificity: 0.500; accuracy: 0.679) cohorts. The AUC of model 1 was lightly superior to that of model 2 in both training and validation sets. However, the difference was not statistically significant (AUCs in the training set: 0.773 vs 0.747, *p* = 0.548; AUCs in validation set: 0.801 vs 0.718, *p* = 0.089) (Table 4 and Fig. 4).

### Performance of model 3

Model 3 showed an excellent predictive performance in the training (AUC 0.889; 95% CI 0.851–0.926; sensitivity: 0.866; specificity: 0.784; accuracy 0.825) and validation (AUC 0.866; 95% CI 0.801–0.928; sensitivity: 0.881, specificity: 0.714, accuracy: 0.798) cohorts. It performed better than model 1 (*p* = 0.001) only in the training set, but not in the validation set (*p* = 0.321) (Table 4 and Fig. 4).

### Clinical utility of the models

**Fig. 5:**
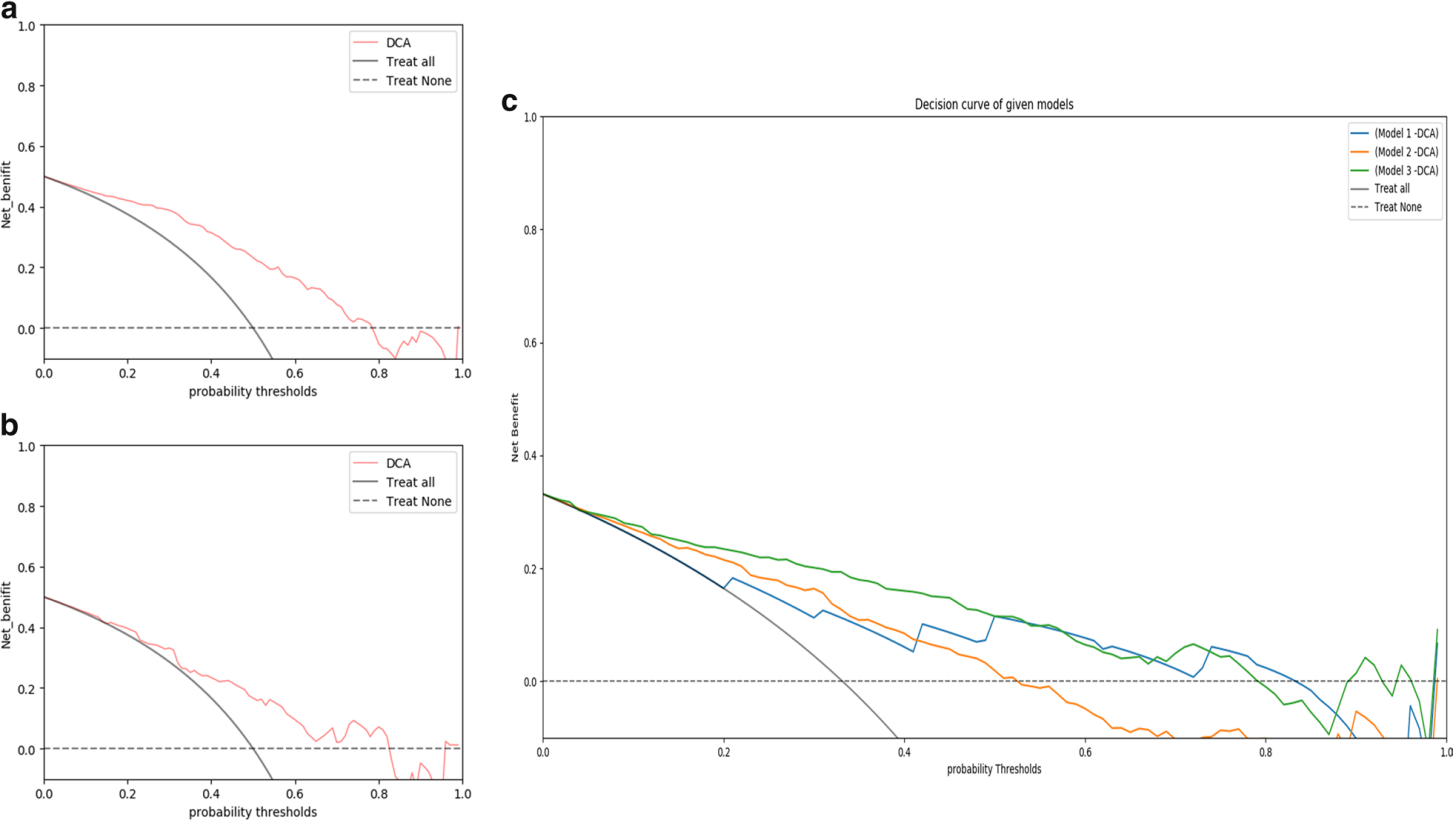
Decision curves analyses (DCA) across all threshold probabilities. The DCA plots the model's net benefit (y-axis) against threshold probability (x-axis) for predicting MTHCC in the entire patients. The “treat all line” assumes all lesions are MTHCC, and the “treat none” line assumes all lesions are non-MTHCC. The DCA of model 1 in training (**a**) and validation sets (**b**); the curve has a higher net benefit than treat-all strategies at a low threshold probability of 10%; (**c**) the decision curve comparison between models 1 (blue), 2 (red), and 3 (green) for all patients

**Fig. 6:**
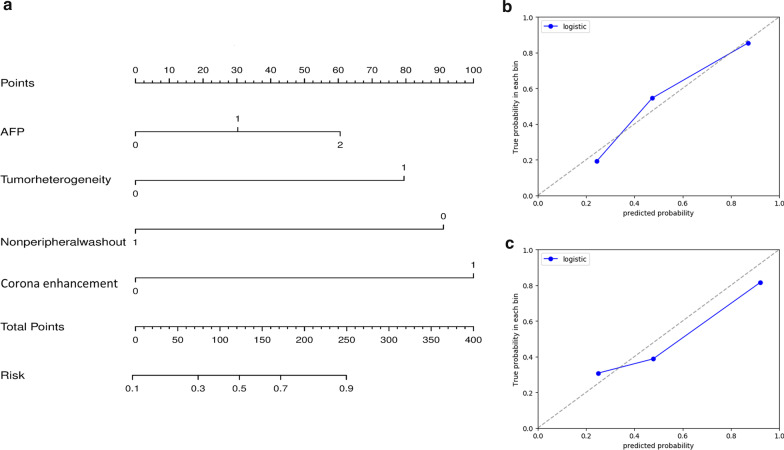
Development of nomogram (**a**) and calibration curves (**b**, **c**) for model 1. It incorporates alpha-fetoprotein levels (AFP), tumor heterogeneity, non-peripheral washout, and corona enhancement. AFP: 0, 1, and 2 represent serum AFP levels of < 20 ng/ml, 20–400 ng/ml, and > 400 ng/ml, respectively. Tumor heterogeneity: 0 and 1 represent homogenous and heterogenous tumor appearance, respectively. 0 represents absence, and 1 represents the presence of washout and APE, respectively. The calibration curves in training (**b**) and validation (**c**) cohorts show the calibration of the nomogram. The diagonal gray dotted line represents the true MTHCC rates, while the blue line demonstrates the predictive performance of the nomogram

The decision curve of model 1 yielded a higher net clinical benefit than the treat-all and treat-none strategy at an MTHCC threshold probability of 10% (which is lower than the reported prevalence of MTHCC) (Fig. 5). A nomogram was constructed for model 1 as it had similar predictive performance to model 3 in the validation cohort and is easier to implement in routine clinical practice (Fig. 6a). It is predicted probabilities closely aligned with the true MTHCC rates in both training (*p* = 0.355) and validation (*p* = 0.364) cohorts (Fig. 6b, c).

## Discussion

MTHCC is an aggressive HCC subtype with poor outcomes. Its identification during patient workup will assist in prognostication and selecting patients who may benefit from more vigorous therapies like radical resection with wide margins or anatomical hepatectomy, as well as strict follow-up schedules. Thus, we developed and validated various predictive models based on MRI to detect the MTHCC subtype preoperatively.

The 2018 EASL clinical practice guideline on HCC management suggests that tumor subtyping has no impact on clinical decision-making [1]. But recent HCC studies indicate that different HCC subtypes exhibit distinct biologic behavior, respond differentially to HCC-directed therapies, and consequently show varied clinical outcomes. For example, the latest WHO classification of HCC [26] includes the MTHCC subtype because of its morphologic peculiarity, clinical relevance, and prognostic implications—it is highly aggressive [6, 27], with greater metastasis rates [28]; has relatively more advanced Barcelona stage [11]; and is an independent indicator of poor clinical endpoints in patients (early and overall recurrence and overall survival) [3, 6, 27]. Therefore, pretherapeutic identification of the MTHCC subtype will be clinically crucial to deciding the best treatment approach and providing individualized patient care.

In our study, serum AFP, tumor heterogeneity, corona enhancement, and absence of the characteristic non-peripheral washout were independent predictors of MTHCC. Similar to previous reports, we found higher levels of serum AFP and greater heterogeneity in the MTHCC subtype [11, 29]. Heterogeneity reflects tumor aggression and cellular and metabolic alterations [30]. Corona enhancement occurs due to the blockage of venous tributaries around the tumor, leading to compensatory peritumoral arterial flow into the surrounding stroma [31]. Also, 12 of our MTHCC did not demonstrate the characteristic washout in both training and validation sets, which was significantly different from the non-MTHCC group (12 vs 3 in the training set, respectively; *p* < 0.001). Washout appearance occurs due to rapid drainage of arterially delivered contrast out of the tumor extracellular compartment and a concomitant reduction in the tumor’s portal supply [32]. Hypoxia-inducing proteins and other pro-angiogenic factors lead to hemodynamic alterations in the MTHCC [4, 33]. Washout disappearance may be due to a reduced outflow of arterially delivered blood or improved portal supply. On univariate analysis, more MTHCCs showed intratumor arteries compared to non-MTHCCs. This is in keeping with findings by Rhee et al., which showed that intratumor arteries are significant predictors of MTHCC [14]. Overall, model 1 showed a good performance in predicting the MTHCC subtype.

Four vital radiomics features (AP-derived glcm_Contrast, PVP-based Skewness, and T2W images-based gldm_DependenceVariance and glszm_ Small AreaEmphasis) associated with MTHCC were obtained from model 2. The MTHCC group demonstrated higher glcm_Contrast, which might result from more intratumor arteries seen in the MTHCCs. Higher skewness occurs when a tumor contains regions of different intensities, implying greater tumor heterogeneity in the MTHCC group. Also, intratumor arteries might skew the lesion’s gray-level distribution as areas of contrast-laden blood vessels in a less enhanced tumor background lead to greater skewness [34]. We attribute the T2W-based radiomics features to MTHCC’s histologic architecture. The gldm_DependenceVariance might reflect the area corresponding to the thick macrotrabecular architecture, while the glszm_SmallAreaEmphasis represents the surrounding web-like vascular spaces [35]. Based on these independent features, model 2 also had a good performance in predicting the MTHCC subtype; its AUC being slightly inferior to model 1 in both training (AUC 0.747 vs. 0.773; *p* = 0.548) and validation (AUC 0.718 vs. 0.801; *p* = 0.089) sets. In a previous study, the fusion radiomics signature and the clinicoradiologic model were performed similarly in both cohorts regarding the preoperative prediction of microvascular invasion [36]. Also, the findings of Ma et al. showed that the HCC radiomics signature performed much lower than the clinical factor model in the validation set (AUC 0.681 vs 0.761), which is in keeping with our findings [37]. This could be because the type of segmentation technique, feature selection and reduction methods, modeling approach, and ultimately variation in the imaging parameters all influence the robustness, reproducibility, and performance of the predictive radiomics signature [38].

Although model 3 (the combined radiomics model) performed better than models 1 and 2 in the training cohort (AUC 0.889; sensitivity: 0.866; specificity: 0.784), its performance was not significantly higher than model 1 in the validation cohort (AUC 0.866 vs 0.801; *p* = 0.321). This indicates that model 1 has a similar diagnostic performance comparable to model 3. Likewise, the performance of the clinicoradiologic model and combined radiomics did not differ in the validation cohorts of several other studies on preoperative HCC histologic characterization [36, 37, 39]. In the validation cohort, Nie et al. [22] reported that the clinicoradiologic and combined radiomics models performed similarly in differentiating focal nodular hyperplasia and HCC (AUC 0.769 vs 0.917; *p* = 0.376). Another study found that the two models had the same diagnostic performance in predicting microvascular invasion of HCC in the validation cohort (AUC 0.850 vs 0.943, respectively; *p* = 0.111) [36]. In HCC, building and clinical validation of a radiomics-based model are challenging and currently limited by the need for large datasets, heterogeneity of image acquisition protocols, as well as difficulty in harmonizing study findings [40, 41]. Since there was no added diagnostic benefit of using the radiomics signature and combined radiomics model in the validation cohort compared to the more simple and easily implementable clinicoradiologic model, this study demonstrates that the radiomics model is of limited benefit in the clinical management of patients. The clinicoradiologic model is thus more suited for the everyday clinical management of MTHCC patients.

The prevalence of MTHCC in this study is 33%, which falls within the range of 15% and 35% reported in the literature [11, 27, 29]. According to the clinical utility analysis, model 1 is beneficial in detecting MTHCC at a lower threshold probability of 10%—a threshold lower than the reported prevalence—reiterating its value in assisting clinicians in improving pretherapeutic decision-making. As it is easier and simpler to build than models 2 and 3, it is more suitable to use in routine clinical practice to predict the MTHCC subtype.

This study has some limitations. First, our retrospective study needs to be externally validated by a larger multicenter prospective study. Second, we used only three phases and did not include HBP because not all patients had gadoxetic acid-enhanced MRI; we also have not included T1W images because some lesions had inconspicuous margins making tumor segmentation difficult. DWI is a powerful MR tool for detecting and characterizing liver lesions. Likewise, we did not include DW images as not all patients underwent DWI during workup; the reliability and stability of HCC radiomics features extracted from DW images are further limited by lower spatial resolution, sensitivity to motion, and varying signal-to-noise ratio with different *b* values [42, 43]. Third, we did not use the LI-RADS categorization in this study. The MTHCC has been associated with a tumor in the vein [12]. Our subsequent study will examine the relationship between the LI-RADS and ADC ratio in the MTHCC subtype, as well as the utility of the ADC as a biomarker for tumor recurrence. Forth, to obtain a reasonably large sample size, the study included patients imaged with two scanners and two different contrast agents, which may inevitably affect the reproducibility of segmentation and feature extraction [44]. Nevertheless, we attempted to achieve reliable segmentation and feature extraction by ensuring a good ICC. Finally, we did not incorporate genomics factors related to MTHCC due to the cost of gene assays. We intend to incorporate radiogenomics and evaluate the impact of different imaging protocols on the robustness of the model in subsequent multicentered series.

In conclusion, three models were developed to predict the MTHCC subtype preoperatively. The clinicoradiologic model (model 1) had a good diagnostic performance in predicting MTHCC in training and validation cohorts. It was slightly superior to the fusion radiomics signature (model 2) in both cohorts and similar to the combined radiomics model (model 3) in the validation cohort. Furthermore, the clinicoradiologic model is easier and simpler to build than the fusion radiomics signature and combined radiomics model in clinical work. Thus, it will be helpful in predicting the MTHCC subtype in routine clinical practice.

## Supplementary Information

**Additional file 1** Supplementary material.

## Data Availability

The data analyzed in this study are not publicly available due to patient confidentiality but are available from the corresponding author upon reasonable request.

## Abbreviations

AUC: Area under the curve
DCA: Decision curve analysis
HCC: Hepatocellular carcinoma
ICC: Interclass correlation coefficient
LASSO: Least absolute shrinkage selection operator
MRI: Magnetic resonance imaging
MTHCC: Macrotrabecular hepatocellular carcinoma
ROC: Receiver operating characteristic curves
