# Supplementary material for: Development and validation of MRI-based model for the preoperative prediction of macrotrabecular hepatocellular carcinoma subtype
Source: Insights Imaging. 2022 Dec 21;13:201. doi: 10.1186/s13244-022-01333-1 (PMC9772375; doi:10.1186/s13244-022-01333-1)
Supplement: Supplementary file 1 — Additional file 1 Supplementary material. [file 13244_2022_1333_MOESM1_ESM.docx]

**ELECTRONIC SUPPLEMENTARY MATERIAL**

**Development and validation of MRI-based models for the preoperative prediction of macrotrabecular hepatocellular carcinoma subtype**

**Materials and methods**

**Appendix E1: Assessment of Qualitative MR features**

The following features for each lesion were evaluated: 1) The number and the diameter of tumors on axial T2W images or hepatobiliary phase as tumor boundary is more clear; 2) tumor necrosis which appears as an area of high signal intensity on T2W images that does not take up the contrast on dynamic phases [8] (**supplementary Fig. 1 (A-D)**); 3) intratumor arteries: appearance of vivid arterial tributaries seen running within the tumor in arterial phase [12] (**supplementary Fig. 1 (B**); 4) corona enhancement: defined as AP crescent-like, a wedge-shaped or irregular enhancement that is separable from the tumor margin and becomes iso-intense with liver parenchyma in the delayed phases [13] (**supplementary Fig. 1 (B)**); 5) tumor heterogeneity which refers to mosaic architectural pattern in the tumor due to presence of components having different signal intensities seen in T2W images and /or dynamic phases [14] (**supplementary Fig. 2**); 6) radiologic capsule: defined as a clear thin linear enhancing structure that partially or completely encase the tumor in the portal or delayed phase and/or a low SI rim on precontrast T1W and T2W images [15] (**supplementary Fig. 2**); 7) intratumor hemorrhage which appears as area of high signal intensity in T2W images, fat-suppressed T1W images, in-phase and out-of-phase sequences (**supplementary Fig. 2**); 8) intra-tumor fat which is defined by an area of high signal intensity in in-phase T1W images that demonstrates signal dropout in the opposed phase (**supplementary Fig. 3**); 9) the characteristic late arterial phase hyperenhancement and non-peripheral washout as defined by the LI-RADS 2018 [16] (**supplementary Fig. 4**); 10) rim-like arterial phase enhancement defined as peripheral irregular rim-like enhancement with central hypo-enhancing areas [17] (**supplementary Fig. 4**).

**Appendix E2: Formula for radiomics score**

Radiomics score was calculated for each patient using the following formula:

Radiomics score = a_1_X_1_ + a_2_X_2_ +…+ a_n_X_n_ + b (1)

Where a_n_ is the LASSO regression coefficient of the variable n, X_n ­_is the value of the n-variable determined from the MR image, b is the intercept. Each selected feature was multiplied by its LASSO coefficient, and the products were added up based on the formula.

**Appendix E3: The nomogram**

The nomogram was graphically presented by proportionally converting the regression coefficients of each predictor in the model to a 0- to a 100-point scale. The effect of the variable with the highest coefficient (absolute value) is assigned 100 points. The points are added across independent variables to derive total points, which are converted to predicted probabilities. The Hosmer-Lemeshow test was used to evaluate the calibration curves of the nomogram.

**Results:**

**Appendix E4**

The radiomics score in model 2 was calculated as follow:

The radiomics score = -0.0142 + 0.5061×original_glcm_Contrast [AP] + 0.3152×original_ shape_Flatness [AP] + 0.6996×original_firstorder_Skewness [PVP] + 0.3312×original_gldm_

DependenceVariance[T2WI]-0.3529×original_glszm_SmallAreaEmphasis[T2WI].

There radiomics scores of MTHCC and non-MTHCC were significantly different in both the training [median score (IQR): 0.819 (-0.262 - 1.618) vs -0.896 (-1.853 - 0.203); p< 0.001] and validation cohorts [median score (IQR): 0.734 (-0.263 to 1.450) vs -0.528 (-1.55 to 0.818); p = 0.006].

**Materials and methods**


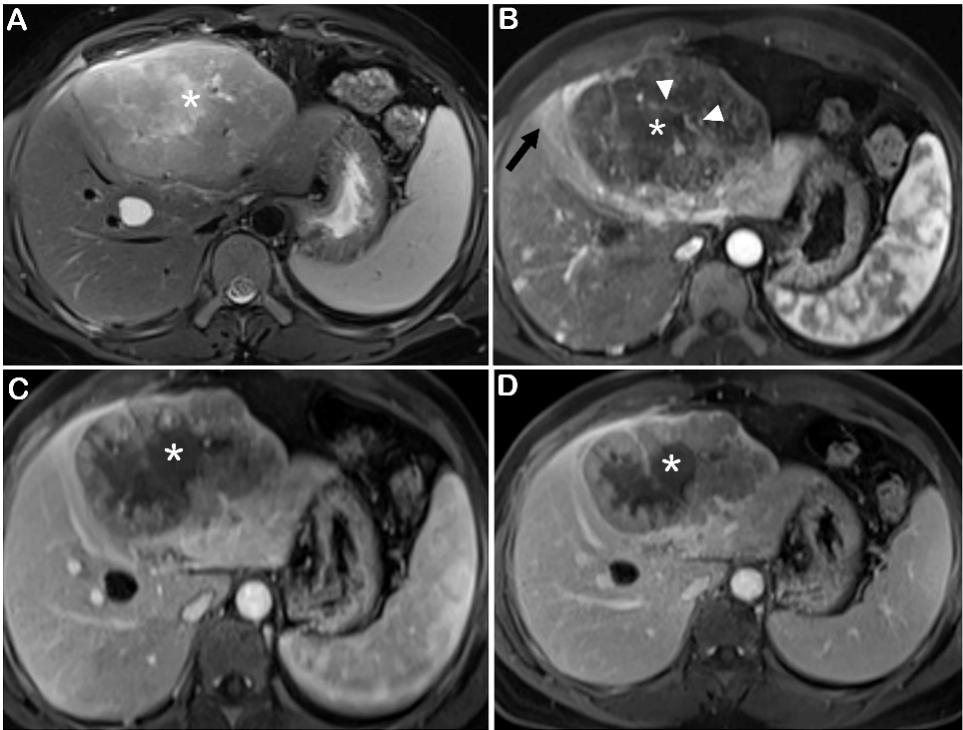


**Supplementary Fig. 1. Tumor necrosis, intratumor arteries, and APPE in an HCC patient with a 12.9 cm tumor (left liver lobe)**. Axial plain fat-saturated T2-weighted (A), arterial phase (B), portal venous phase (C), and delayed phase (D) images showing tumor necrosis (*) as an area of high signal intensity on T2-weighted image (A) and devoid of contrast in arterial (B), portal (C), and delayed phases (D). Intratumor arteries (arrowheads) are vivid arterial tributaries seen in the arterial phase (B). The corona enhancement appears as a wedge-shaped enhancement (black arrow) in the arterial phase (B) that is separable from the tumor margin, which then becomes iso-intense with liver parenchyma in the portal and delayed phases (C and D).


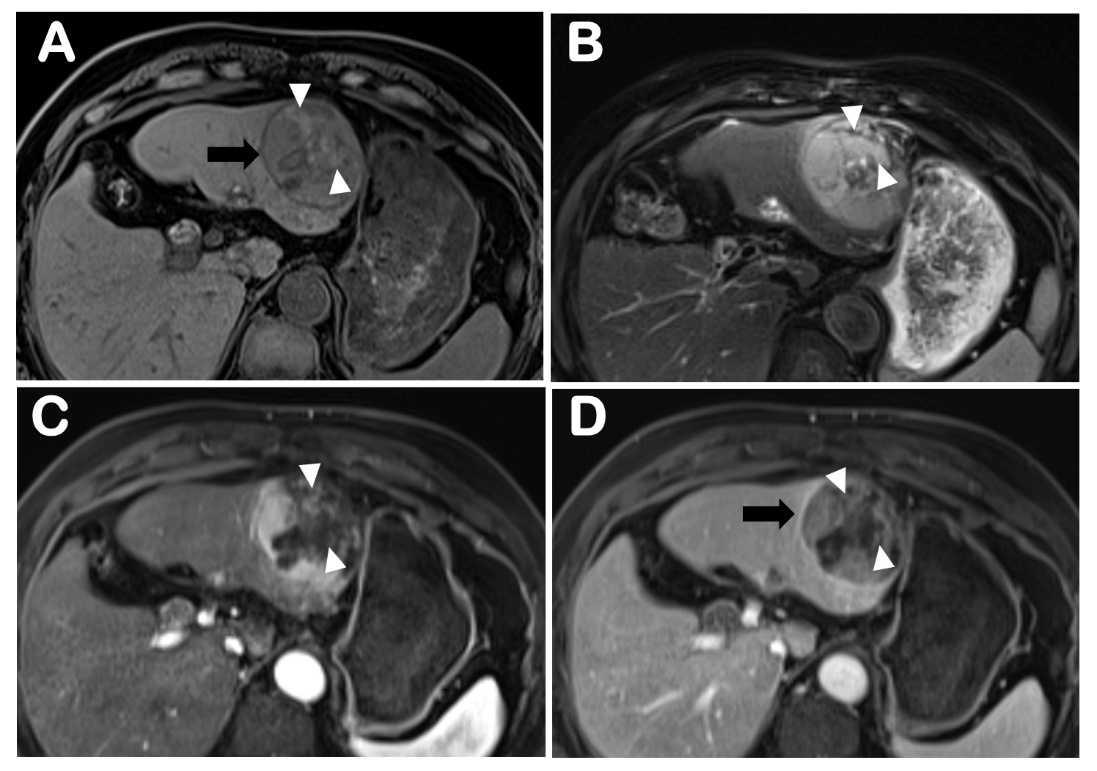


**Supplementary Fig. 2. Tumor heterogeneity, radiologic capsule, and hemorrhage in an HCC subject with 6.5cm mass (left liver lobe)**. Axial plain fat-saturated T1-weighted (A), T2-weighed (B), arterial phase (C) and portal venous phase (D) images show tumor heterogeneity with mosaic pattern of different signal intensities due to the presence of multiple components on T2W images and dynamic phases. Intratumor hemorrhage (arrowheads) appears hyperintense on T1WI (A), slightly hyperintense on T2WI (B) and no enhancement on arterial (C) and portal venous phase (D) images. The lesion has a clear thin linear radiologic capsule (black arrows), which appears hypointense in T1WI (A) and enhanced in the portal venous phase (D).


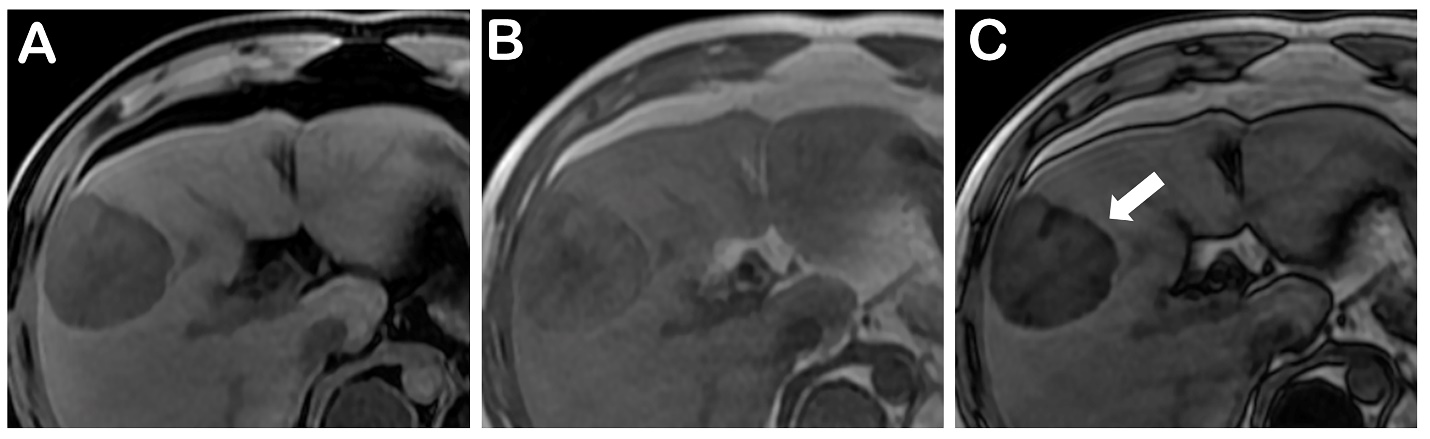


**Supplementary Fig. 3. Intra-tumor fat in an HCC patient with a 16.5 cm tumor (right liver lobe)**. Axial fat-saturated T1-weighted (A), and in-phase (B) and opposed-phase (C) images show intratumor fat, which appears as a drop of signal in the lesion (white arrow) relative to liver parenchyma on opposed-phase (C) compared to in-phase (B)


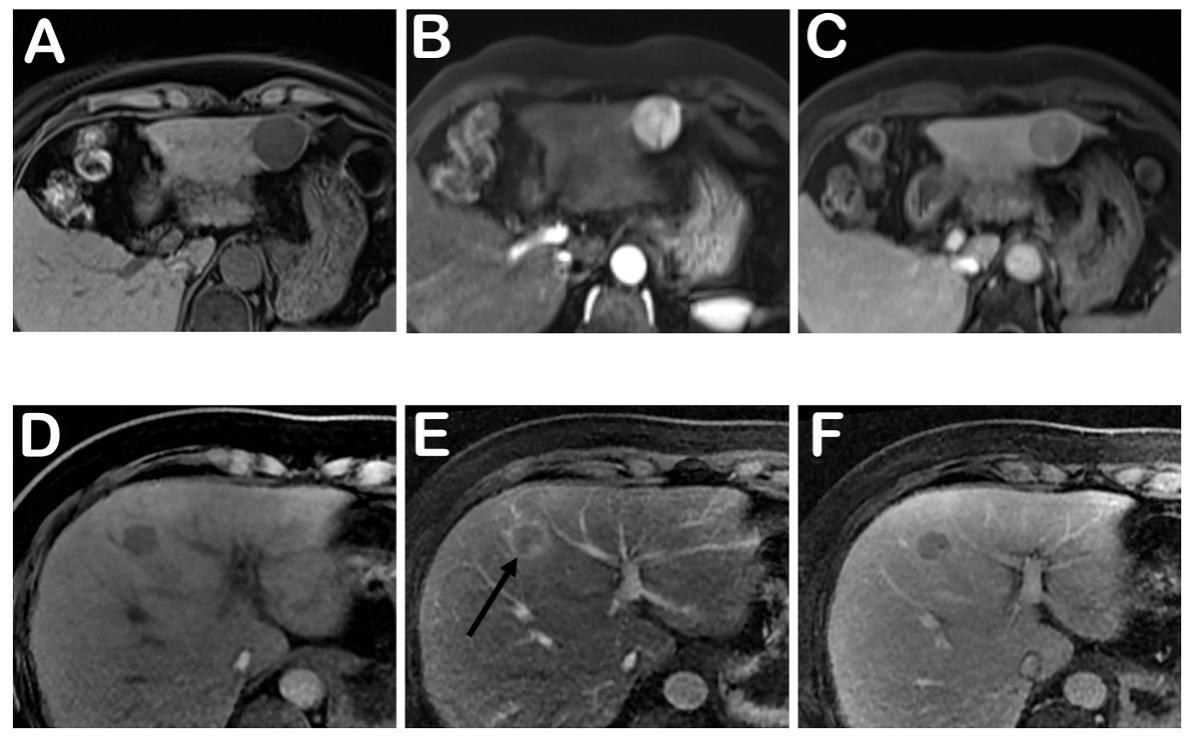


**Supplementary Fig. 4. Representative MR images in two patients showing non-rim and rim arterial phase hyperenhancement** on axial fat-saturated T1-weighted (A and D), arterial phase (B and E) and portal venous phase (C and F) images. In the MR images of the first patient, the lesion shows non-rim arterial phase hyperenhancement (B), and non-peripheral washout in the portal venous phase (C). But the MR images of the second patient show rim (black arrow) arterial phase hyperenhancement (E) and non-peripheral washout in portal venous phase (F). Both lesions showed low intensity on T1-weighted images (A and D).

**Results**


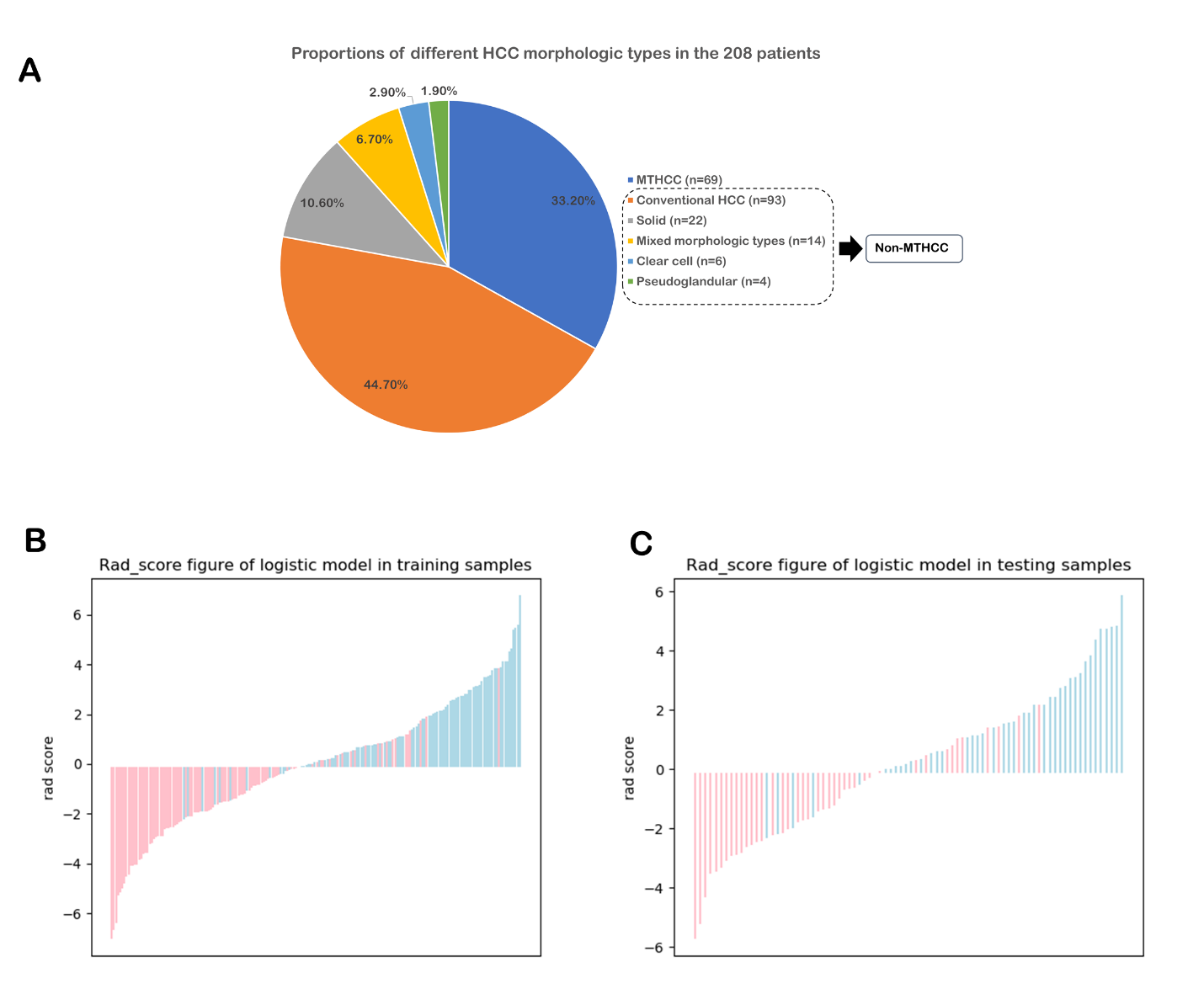
 **Supplementary Fig. 5. The pie chart illustrates the proportions of MTHCC and non-MTHCC subtypes in the 208 HCC patients (A)**. **The bar charts show the radiomics score in training (A) and validation (B) cohorts.** The blue bars represent scores for MTHCC, while the red bars represent the scores for non-MTHCC. The radiomics score makes a correct prediction when the blue bar is positive or the red bar is negative, and not vice versa

| Supplementary Table 1: Number of features extracted from each ROI. | | | | | |
| --- | --- | --- | --- | --- | --- |
| **Category** | **Texture Feature** | **Original features** | **LoG^*^** | **Wavelet^*^** | **Total** |
| Stochastic | Shape-based | 14 | - | - | 14 |
| First-order | Histogram based | 18 | 36 | 144 | 198 |
| Second-order | GLCM | 24 | 48 | 192 | 264 |
|  | GLRLM | 16 | 32 | 128 | 176 |
|  | GLSZM | 16 | 32 | 128 | 176 |
|  | NGTDM | 5 | 10 | 40 | 55 |
|  | GLDM | 14 | 28 | 112 | 154 |
|  | | | |  | 1037 (total) |
| LoG Laplacian of Gaussian and wavelet transform= higher-order features; GLCM grey level cooccurrence matrix; GLRLM grey-level run-length matrix; GLSZM grey-level size zone matrix; NGTDM neighborhood grey tone difference matrix; GLDM grey-level dependence matrix. | | | | | |
